# Lipid-Based Nanovesicles for Simultaneous Intracellular Delivery of Hydrophobic, Hydrophilic, and Amphiphilic Species

**DOI:** 10.3389/fbioe.2020.00690

**Published:** 2020-07-03

**Authors:** Antonella Zacheo, Luca Bizzarro, Laura Blasi, Clara Piccirillo, Antonio Cardone, Giuseppe Gigli, Andrea Ragusa, Alessandra Quarta

**Affiliations:** ^1^CNR NANOTEC—Institute of Nanotechnology, c/o Campus Ecotekne, Lecce, Italy; ^2^Dipartimento di Scienze Biomolecolari (DISB), University of Urbino Carlo Bo, Urbino, Italy; ^3^CNR, Institute for Microelectronics and Microsystems, Lecce, Italy; ^4^Institute of Chemistry of OrganoMetallic Compounds—ICCOM, Italian National Council of Research—CNR, Bari, Italy; ^5^Department of Mathematics and Physics E. de Giorgi, University of Salento, Campus Ecotekne, Lecce, Italy; ^6^Department of Biological and Environmental Sciences and Technologies, University of Salento, Lecce, Italy

**Keywords:** nanovesicle, nanoparticle, doxorubicin, SN-38, breast cancer, lipidomic analysis

## Abstract

Lipid nanovesicles (NVs) are the first nanoformulation that entered the clinical use in oncology for the treatment of solid tumors. They are indeed versatile systems which can be loaded with either hydrophobic or hydrophilic molecules, for both imaging and drug delivery, and with high biocompatibility, and limited immunogenicity. In the present work, NVs with a lipid composition resembling that of natural vesicles were prepared using the ultrasonication method. The NVs were successfully loaded with fluorophores molecules (DOP-F-DS and a fluorescent protein), inorganic nanoparticles (quantum dots and magnetic nanoparticles), and anti-cancer drugs (SN-38 and doxorubicin). The encapsulation of such different molecules showed the versatility of the developed systems. The size of the vesicles varied from 100 up to 300 nm depending on the type of loaded species, which were accommodated either into the lipid bilayer or into the aqueous core according to their hydrophobic or hydrophilic nature. Viability assays were performed on cellular models of breast cancer (MCF-7 and MDA-MB-231). Results showed that NVs with encapsulated both drugs simultaneously led to a significant reduction of the cellular activity (up to 22%) compared to the free drugs or to the NVs encapsulated with only one drug. Lipidomic analysis suggested that the mechanism of action of the drugs is the same, whether they are free or encapsulated, but administration of the drugs by means of nanovesicles is more efficient in inducing cellular damage, likely because of a quicker internalization and a sustained release. This study confirms the versatility and the potential of lipid NVs for cancer treatment, as well as the validity of the ultrasound preparation method for their preparation.

## Introduction

Lipid-based vesicles are the most commonly used carriers for drug delivery in nanomedicine thanks to their ease of preparation, excellent biocompatibility, and structural plasticity. They are conventionally defined as spherical vesicles characterized by an outer bilayer of lipids with an internal aqueous cavity. Since the first description of liposomal formulation, almost 60 years ago, many types of lipid vesicles have been developed for applications in various areas, including drug and gene delivery (Grimaldi et al., 2016). Some of them have been already approved for clinical use in cancer chemotherapy as carriers of pharmaceuticals, such as doxorubicin, irinotecan, cisplatin, and placlitaxel (Bulbake et al., 2017). Other formulations are currently under clinical evaluation for the delivery of drugs in cancer and other diseases.

The considerable research efforts in this area are motivated by the numerous benefits provided by the use of liposomes, including their ability to host both lipophilic and hydrophilic molecules, the presence of an aqueous core that can accommodate large compounds, the high affinity with the cell membrane that facilitates their internalization, the biodegradability and the negligible toxicity of the vesicle components. Indeed, they are generally composed of natural lipids; among them, phospholipids, such as phosphatidylethanolamine and phosphatidylcholine, and sterols, such as cholesterol, have been used as components of the bilayer (Li et al., 2019).

The amount of cholesterol and the length and saturation of the hydrocarbon chains of the phospholipids affect the rigidity and the stability of the bilayer, and in turn the capability of the NVs to host and release drugs/biomolecules (Monteiro et al., 2014). On the other hand, functionalization of the hydrophilic heads of the lipids with polymers or biomolecules, provides additional features to the vesicle surface, thus shaping their interaction with blood components, tissues, and the immune system *in vivo* (Riaz et al., 2018). For instance, surface coating with polyethyleneglycol (PEG) polymer chains has been demonstrated to ameliorate the colloidal stability of the liposomes and hence their capability to elude the immune system and prolong the circulation time (Nkanga et al., 2019).

Depending on the preparation method, it is possible to tune both size and lamellarity of lipid-based vesicles. Conventional synthetic approaches, which include film hydration, reverse-phase evaporation, and detergent dialysis, can produce multilamellar vesicles; however, they suffer from high size polydispersity and low reproducibility (Kraft et al., 2014). On the other hand, methods based on the application of mechanical forces, such as sonication and homogenization, allow for a finer control of the vesicle's size and uniformity. More recently, microfluidic technologies have been applied to the synthesis of uniform lipid vesicles (Carugo et al., 2016).

In a previous work, we also designed a microfluidic system for the preparation of lipid nanovesicles with highly homogeneous size distribution and good reproducibility (Zacheo et al., 2015). However, this method suffers from low particle yield per volume and long preparation times. In the present work, NVs with a similar lipid composition were prepared using the more versatile ultrasonication method (Klingler et al., 2015; Salvi and Pawar, 2019). NVs were synthesized combining two phospholipids [1-lauroyl-2-hydroxy-*sn*-glycero-3-phosphocholine (LPC) and 1,2-dilauroyl-*sn*-glycero-3-phosphocholine (DLPC)] with a ceramide [*N*-lauroyl-D-erythro-sphingosine (CER)] and cholesterol (CHOL). The molar ratio of the lipids was set in order to resemble the lipid composition of vesicles naturally released by cells, that are typically rich in cholesterol, sphingolipids, and phospholipids (Antimisiaris et al., 2018; Skotland et al., 2019). To demonstrate the high versatility of this system, several compounds with different physico-chemical properties (i.e., hydrophilic, hydrophobic, and amphiphilic molecules) and dimensions (with molecular weight ranging from few hundreds Da to hundreds kDa) were encapsulated into the NVs. More specifically, inorganic nanocrystals, such as quantum dots (QDs) and magnetic nanoparticles (MNPs), an organic amphiphilic fluorophore (i.e., DOP-F-DS), the transferrin protein, and two anticancer drugs (i.e., doxorubicin and SN-38) were loaded either into the bilayer or into the core.

Doxorubicin (DOXO) is commonly employed in the chemotherapy of several solid tumors and leukemia, especially in its lipid-based formulation, the first one to be approved for clinical use (Barenholz, 2012). On the other hand, SN-38, namely 7-ethyl-10-hydroxy camptothecin, is a topoisomerase I inhibitor used in cancer therapy against solid tumors such as colon carcinoma, breast, ovarian, and pancreatic cancers (Wallin et al., 2008). It is characterized by low water solubility and poor stability in the blood stream. Therefore, high doses are needed in clinical therapy, also causing serious toxic side-effects in patients. As such, a liposomal formulation of this drug would provide its beneficial protection until reaching the target site and a prolonged circulation time of the active ingredient (Fang et al., 2018).

The prepared NVs were fully characterized and the thermal stability, size, morphology, surface charge, and colloidal stability over time assessed. The efficiency of the drug encapsulation and the effectiveness of the system as anti-cancer carrier were tested on selected human cancer cellular lines**—**MCF-7 and MDA-MB-231. Furthermore, the effect of the two drugs, either free or loaded into the NVs, on the cancer cells was studied via lipidomic analysis. This type of study is gaining increasing attention as it can provide valuable information about the changes that occur upon a particular stimulus, such as drug administration (Zhao et al., 2015; Perrotti et al., 2016). Lipids are the mayor constituents of the cellular membrane and they are also involved in many biological processes strongly related to carcinogenic pathways, such as transformation, progression, and metastasis, and their composition is altered in many neoplastic diseases (Giudetti et al., 2019). Nuclear magnetic resonance (NMR) analysis of the variation in the lipid composition upon drug treatment can thus provide valuable information about efficacy and progression of the treatment.

## Materials and Methods

**Figure 1:**
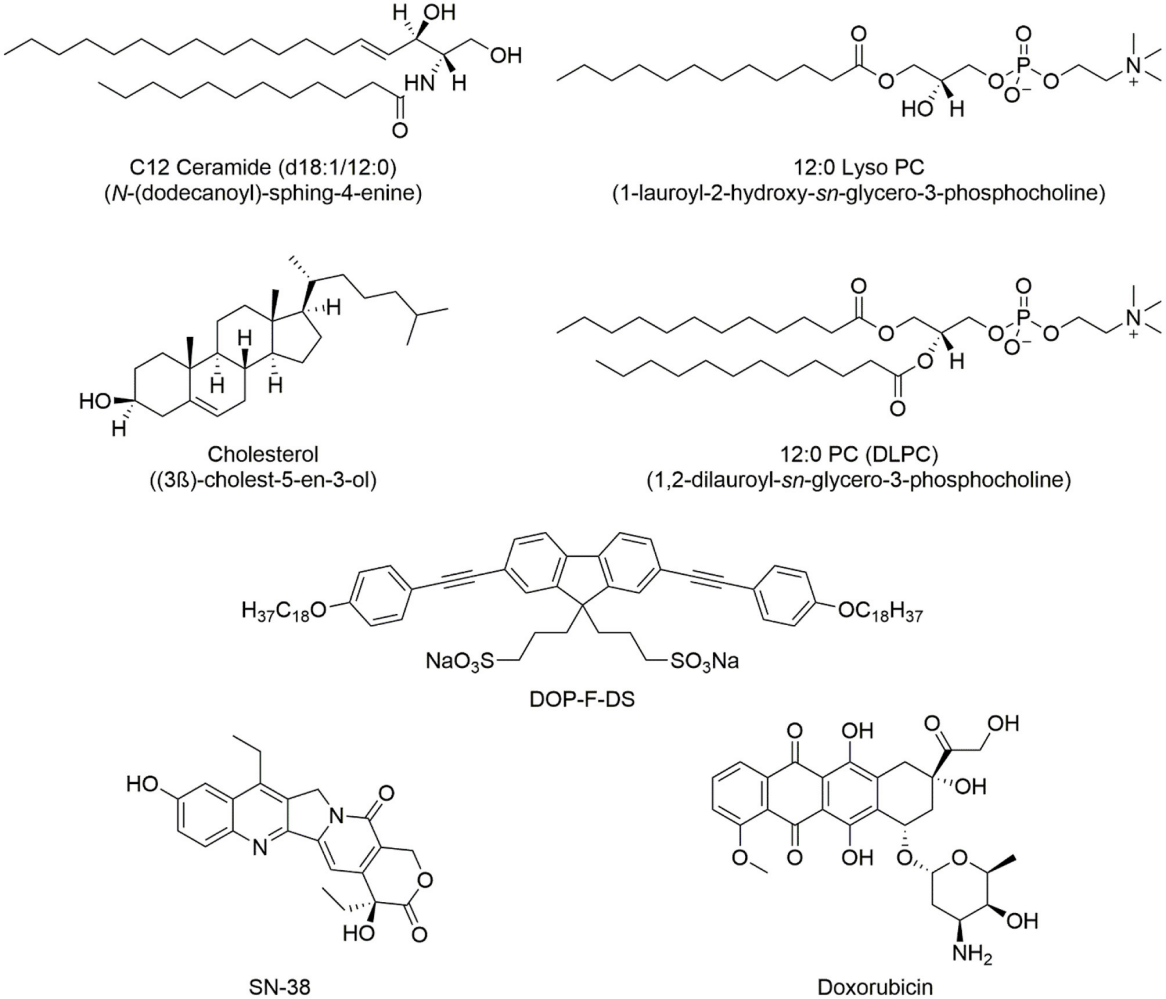
Chemical structure of the lipids used in the synthesis of the NVs and the encapsulated organic molecules, (i.e., the DOP-F-DS fluorophore and the two drugs used in cellular study).

1,2-Dilauroyl-*sn*-glycero-3-phosphocholine (DLPC, 12:0), 1-lauroyl-2-hydroxy-*sn*-glycero-3-phosphocholine (LPC, 12:0), cholesterol (ovine wool >98%, CHOL), and *N*-lauroyl-D-erythro-sphingosine (saturated ceramide, d18: 1/12: 0, CER) were purchased from Avanti Polar Lipids. The molecular formulas of the lipids are reported in Figure 1. Doxorubicin and transferrin-TRITC were purchased from Sigma-Aldrich and ThermoFisher, respectively. SN-38 was a kind gift from Ospedale Oncologico Giovanni Paolo II in Bari, Italy. The organic fluorophore DOP-F-DS (Figure 1) was prepared as previously reported in the literature (Cardone et al., 2012). CdSe/ZnS QDs and iron oxide MNPs were prepared according to the procedures reported in the literature (Dabbousi et al., 1997; Hyeon et al., 2001). The two cell lines, namely MCF-7 and MDA-MB-231, were obtained from ATCC.

### Synthesis and Characterization of the Nanovesicles

A mixture of lipids consisting of DLPC, CHOL, LPC, and CER with respective ratios of 4.5:4:1:0.5 was used for the synthesis of the vesicles (final concentration 5 mM in 5 mL of chloroform). The phospholipid solution was stirred for 1 h with the cap closed to prevent any loss of solvent. Subsequently, the cap was removed and the solution left stirring overnight at room temperature to evaporate the solvent. Phosphate saline buffer (PBS) was then added and the solution was sonicated for 4 min at a power of 25 W and at 20 kHz frequency. During the sonication the vial was kept into an ice bath to prevent overheating of the solution.

To prepare the vesicles containing transferrin-TRITC and the blue-emitting DOP-F-DS organic fluorophore, 150 μL of DOP-F-DS (1 mg/mL in DMSO), and 50 μL of transferrin-TRICT (5 mg/mL in PBS) were respectively employed. To load SN-38 into the liposomes, 200 μL of the drug dissolved in DMSO (200 μM) were added to the phospholipids in chloroform and the mixture was left under vigorous stirring for 60 min. Subsequently, the vesicles were prepared following the protocol already described. The hydrophobicity of the SN-38 molecule facilitates its intercalation within the double phospholipidic layer of the liposomes. In the case of doxorubicin, the drug dissolved in PBS (85.8 μM final concentration) was added to the dry phospholipid film. The NVs were then prepared as already described. To load both drugs into the vesicles, solutions at the same concentration as before were used. The NVs were then prepared according to the standard conditions but sonicated for longer time (6 min). To encapsulate the inorganic nanoparticles within the vesicles, few microliters of a chloroform solution of either QDs or MNPs were added to the starting lipid mixture (see Table 1 for details).

**Table 1:** Conditions employed for the preparation of the nanovesicles (type of loaded species, volume, concentration, and solvent used) and their application in this study.

**Loaded species**	**Volume**	**Concentration**	**Application**
DOP-F-DS	15 μL	8.4 mM in DMSO	Uptake analysis
Transferrin-TRITC	25 μL	13 mM in PBS	Uptake analysis
DOP-F-DS	15 μL	8.4 mM in DMSO	Uptake analysis
+ Transferrin-TRITC	25 μL	13 mM in PBS	
Doxorubicin	25 μL	17.2 mM in PBS	Antitumor activity
SN-38	25 μL	2.5 mM in DMSO	Antitumor activity
Doxorubicin	25 μL	17.2 mM in PBS	Antitumor activity
+ SN-38	25 μL	2.5 mM in DMSO	
Surfactant-coated MNPs	25 μL	3 μM in CHCl_3_	Magnetic responsiveness
Surfactant-coated QDs	15 μL	6 μM in CHCl_3_	Uptake analysis

The solution containing the formed vesicles was then transferred into a dialysis tube (50 kDa MWCO), placed into 2 L of PBS, and kept under stirring at 4°C for 48 h. After purification, the nanovesicles were fully characterized.

Size and morphology of the lipid vesicles were monitored through transmission electron microscopy (TEM) and dynamic light scattering (DLS) measures. Low-magnification TEM images of the nanovesicles were recorded on a JEOL Jem1011 microscope operating at an accelerating voltage of 100 kV. DLS and zeta potential measurements were performed in PBS at 25°C using a Zetasizer Nano ZS90 (Malvern Instruments Ldt) equipped with a 4.0 mW He–Ne laser operating at 633 nm and with an avalanche photodiode detector.

Thermal stability was assessed by thermogravimetric analysis (TGA) using SDT Q600 equipment (TA Instruments) with a heat ramp of 5°C/min and an air flow rate of 100 mL/min. After synthesis of the NVs, the solvent was evaporated and the analysis was performed on the residual dry matter. Control analyses were also done on the free lipids, which were mixed in chloroform at the same molar ratio before removing the solvent.

Encapsulation efficiency (*EE*) and release profile of the encapsulated drugs were determined spectrophotometrically with a fluorescence spectrometer (Cary Eclipse) measuring the fluorescence intensity of the drugs. A calibration curve at known concentrations of the molecules was first prepared. The amount of free drug dissolved in the dialysis medium (PBS), after purification, was measured and the *EE*, expressed as percentage of encapsulated molecules over the total, was calculated according to the formula:

$$\begin{array}{l} EE\;\left(\%\right)=\frac{\left(Initial\;drug\;amount-free\;drug\;in\;the\;dyalisis\;medium\right)}{Initial\;drug\;amount}\;\times 100 \end{array}$$

To determine the drug release profile, samples were kept at 37°C at two pH (4.5 and 7.4) for different time lengths (6, 24, 48, 96, and 120 h). The vesicles were then pelleted and the amount of drug in the supernatant quantified. The released amount at each time point, expressed as percentage over the total encapsulated, was estimated according to the formula:

$$\begin{array}{l} Released\;amount\;\left(\%\right)=\frac{released\;drug\;concentration}{encapsulated\;drug\;concentration}\times 100 \end{array}$$

### Cellular Studies

#### Viability Assays

Two human cell lines of mammary carcinoma, namely MCF-7 and MBA-MB-231, were used. The cells were grown in DMEM medium supplemented with 10% of fetal bovine serum (FBS), 2 mM glutamine, 100 IU/mL of penicillin, and 100 μg/mL of streptomycin, and were cultured in an incubator at 37°C in a humidified atmosphere with 5% CO_2_.

Two viability assays were performed, namely MTT and Trypan blue assays. In detail, 5 × 10^4^ cells suspended in 200 μL of culture medium were seeded into each well of 96 multiwell plates. After 24 h incubation at 37°C, the nanovesicles were added to the wells at a defined concentration (each point triplicated) and the cells were kept under incubation for three different times, 24, 48, and 120 h.

In the case of the MTT assay, at the end of the incubation time, the medium was removed, the cells were washed twice with PBS, and 200 μL of fresh serum-free medium containing 1 mg/mL MTT were added to each well. After 3 h of incubation at 37°C, the medium was discarded from the wells and 200 μL of DMSO were added to dissolve the formazan salts. The plate was stirred for a few minutes at ambient temperature and the absorbance of the solution at 570 nm was measured on a microplate reader. To determine the percentage of cell viability, the treated samples were compared to the control samples according to the equation:

$$\begin{array}{l} Cell\;viability\;\left(\%\right)=\frac{Absorbance\;of\;the\;NVs-containing\;sample}{Absorbance\;of\;the\;control\;sample}\times 100 \end{array}$$

To check the presence of a synergic effect by the combined delivery of the two drugs, the combination index (CI) was also determined according to the following formula (Chou and Talalay, 1984):

$$\begin{array}{l} CI_{50}=\frac{C_{A}}{{IC_{50}}_{A}}+\frac{C_{B}}{{IC_{50}}_{B}} \end{array}$$

where C_A_ and C_B_ are the concentrations of drugs A and B co-loaded into the NVs that were used to achieve the IC_50_ effect. IC_50A_ and IC_50B_ are the concentrations of the single drugs used to achieve the same effect on cell viability.

In the case of the Trypan blue assay, at the end of the incubation time, the medium was removed, the cells were washed twice with PBS, and 100 μL of PBS containing Trypan blue diluted 1/10 were added to each well. Soon after, the plates were analyzed under the microscope and the number of viable and dead cells was counted. The cell viability was determined as follows:

$$\begin{array}{l} Cell\;viability\;\left(\%\right)=\frac{Number\;of\;viable\;cells}{Number\;of\;total\;cells}\times 100 \end{array}$$

#### Lipidomic Analysis

MDA-MB-231 cells (1 × 10^8^ cells/well, three replicates each) were treated with the empty nanovesicles, with the free drugs alone or in combination, and with the nanovesicles loaded with either one or both drugs. After 24 h incubation, cells were quenched and the lipid fraction extracted according to previously published procedures (Sündermann et al., 2016). The extracts were dried under a gentle nitrogen flow, dissolved in 600 μL of deuterated chloroform containing 0.03% of tetramethylsilane (TMS), used as the reference, and transferred into 5 mm NMR tubes.

Spectra were acquired on a Varian INOVA spectrometer (Varian Inc., CA) operating at 499.792 MHz and equipped with a OneNMR Probe-PT (Agilent). Acquisition parameters were set as follows: *s2pul* pulse sequence, 128 scans, 25°C, pulse angle 45°, sw 8012.8 Hz, relaxation delay 1 s, acquisition time 2.045 s, np 32,768, complex points 16,384, observed pulse 4.85, calibration *pw90* 9.70 μs. The resulting spectra were Fourier-transformed, phase corrected, and calibrated to the signal of TMS. The region between 0.5 and 5.5 ppm was binned into 0.02 ppm-wide buckets (equal to 238 variables) and exported to a csv file containing the data for all samples. The obtained matrix was normalized to the total sum and Pareto scaled before multivariate statistical analysis.

#### Confocal Microscopy Imaging

For cell imaging analysis, 10^5^ cells were seeded onto a coverslip placed into each well of a six-well plate. After 24 h, the fluorescent nanovesicles were added to each well and the cells were incubated for the settled time (6, 24, and 48 h). Then, they were washed with PBS and fixed with 4% paraformaldehyde prior to be imaged by means of a Leica confocal microscope (TCS-SP5, Leica, Mannheim, Germany) equipped with an Argon laser source (excitation at 488 nm for green emitting QDs, at 545 nm for Transferrin-TRITC and Phalloidin-TRITC) and a pulsed laser (excitation at 405 nm for DOP-F-DS and DAPI).

For the colocalization study, MDA-MB-231 cells were seeded as already described and then incubated with the NVs loaded with transferrin-TRITC for the settled time (6, 24, and 48 h). Then, cells were washed with PBS and stained with Lysotracker green (Molecular probes) according to the manufacturer protocol. The samples were imaged by means of the confocal laser scanning microscopy (CLSM).

#### Statistical Analysis

All data represent the average value of at least three independent experiments, unless otherwise specified. Normally distributed data was compared with a two-tailed Student's *t*-test using GraphPad Prism software (version 6.0). The bars in graphs represent mean ± S.D. values. Differences were considered significant when the *p*-value was < 0.05. Multivariate statistical analysis in the lipidomic study was performed with SIMCA 14.1 software (MKS Umetrics, Malmö, Sweden) using pairs of classes in each analysis (control vs. free drugs; control vs. encapsulated drugs; free drugs vs. encapsulated drugs; control vs. empty NVs). Orthogonal partial least-squares discriminant analysis (OPLS-DA) was performed using the NMR buckets as variables, the samples as observations, and the formulations (drugs-loaded NVs, empty NVs, free drugs, no drugs) as classes. Cumulative $R_{\text{X}}^{2}$ and $R_{\text{Y}}^{2}$ were used as parameters for describing the goodness of the fit.

## Results and Discussion

### Synthesis and Characterization of Lipid Nanovesicles

NVs were prepared using the above-mentioned compounds according to the molar ratio DLPC/CHOL/LPC/CER 4.5:4.0:1.0:0.5. The lipid composition of these vesicles was designed to mimic the structure of the extracellular vesicles released physiologically by the cells having important roles in intercellular communication and in the onset and transmission of diseases (Trajkovic et al., 2008; De Toro et al., 2015; Zhang et al., 2015; Sarko and McKinney, 2017; Skotland et al., 2017; Steinbichler et al., 2017). This affinity should in turn facilitate the interaction with the cell membrane and promote cellular uptake.

Simultaneous encapsulation of hydrophobic, hydrophilic, and amphiphilic species inside the NVs was achieved, with the hydrophobic molecules being located into the lipid bilayer while the hydrophilic ones into the aqueous core. An intermediate behavior would be expected in the case of amphiphilic molecules. Several parameters, such as the molar ratios of the three components, sonication time and power together with the volumes of PBS were modulated to obtain particles with an average size around 100 nm and a good size distribution. Particles with the desired characteristics were obtained by sonicating at 25 W for 4 min.

**Figure 2:**
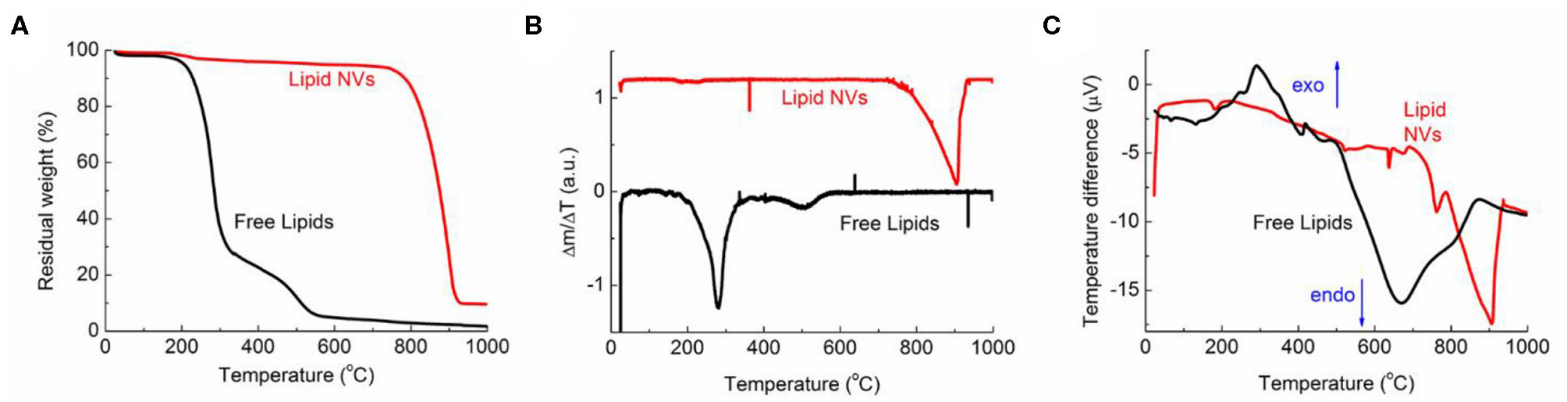
TGA and DSC data for NVs (red curve) and free lipids (black curve). **(A)** Percentage weight, **(B)** first derivative curves, and **(C)** DSC data.

To study the thermal stability of the developed nanovesicles, TGA analysis was performed and the results are reported in Figure 2. As a comparison, the test was also performed on the free lipid components. The weight loss percentage (Figure 2A) showed a completely different behavior between the nanovesicles and the free lipids; for the lipids, in fact, complete degradation was observed for 200 < T < 600°C. Such degradation took place in two main steps, the first corresponding to a weight loss of about 70% (T < 350°C) while the second of about 25% (T > 350°C). The curve of the first derivative (Figure 2B) highlighted additional smaller steps at lower temperatures.

On the other hand, the data showed a much higher stability of the nanovesicles, as very small weight loss (about 5%) was observed for T < 700°C, while their degradation took place between 700 and 900°C. This behavior was confirmed by the first derivative curve.

Significative differences between the nanovesicles and the free lipids could be also observed in the DTA curves (Figure 2C). For the free lipids, in fact, an exothermic peak was observed for 200 < T < 400°C; this can be due to the combustion of some organic fragments/molecules, in agreement with the literature (López-González et al., 2015). This peak is not present in the corresponding curve of the nanovesicles, indicating that the formation of the vesicle structure makes the whole system more thermally stable. The endothermic peaks, corresponding to the full chemical degradation of the molecules/nanovesicles, can be observed in both curves, but at different temperatures, (i.e., between 500 < T < 800°C and 770 < T < 900°C for free lipids and nanovesicles, respectively). Literature data already report of increased thermal stability of the vesicle structure in comparison to the free compounds (Pinilla et al., 2019). This is likely due to the different special arrangements of the molecules; indeed, stronger interactions can take place when lipids are confined in a layer, leading to a more thermally stable system.

Different molecules were loaded into the vesicles, as listed in Table 1, either in the lipid bilayer or the aqueous core, according to their physico-chemical characteristics. The same sonication power was used for all experiments, although a longer duration (6 min compared to the four used for all the other cargos) was necessary only when both drugs were encapsulated simultaneously.

The procedure was slightly modified depending on the hydrophobic/hydrophilic/amphiphilic nature of the species to be introduced into the vesicles. In the case of the amphiphilic DOP-F-DS fluorophore or the hydrophobic antineoplastic SN-38 drug, the species were added to the lipid suspension. On the other hand, when hydrophilic molecules, such as the fluorescent transferrin or doxorubicin, had to be encapsulated, they were dissolved in PBS and then added to the lipid dry film before sonication.

**Figure 3:**
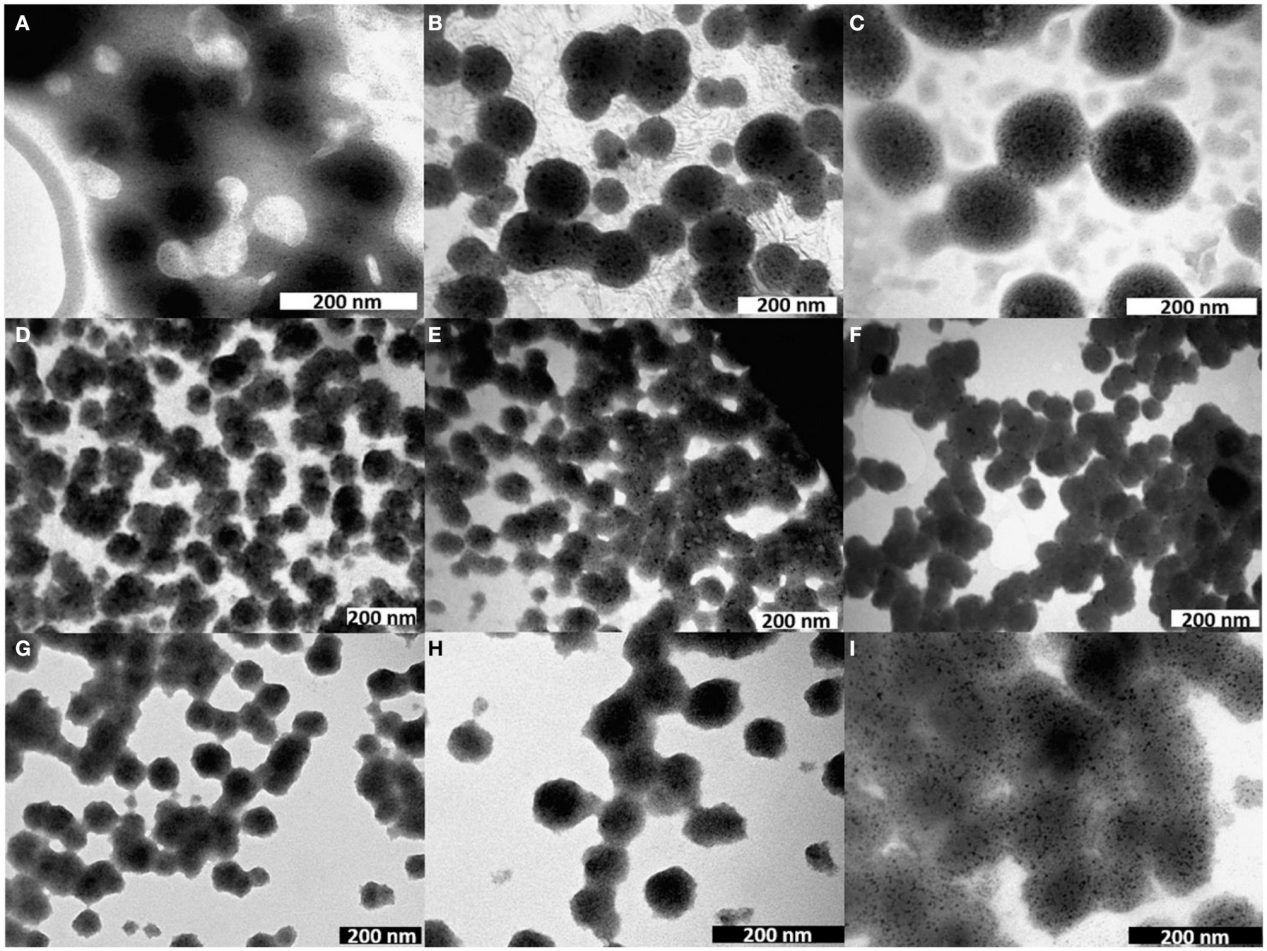
TEM images of nanovesicles either empty **(A)** or loaded with **(B)** QDs, **(C)** MNPs, **(D)** DOP-F-DS, **(E)** transferrin-TRITC, **(F)** DOP-F-DS, and transferrin-TRITC, **(G)** doxorubicin, **(H)** SN-38, and **(I)** both drugs.

The vesicles were analyzed by different techniques to determine their morphology, size, charge and thermal stability. Figure 3 shows the TEM images of the empty NVs and of the NVs loaded with different species, such as hydrophobic QDs, MNPs, the amphiphilic fluorophore DOP-F-DS, transferrin-TRITC, both DOP-F-DS and transferrin-TRITC, doxorubicin, SN-38, and both drugs.

The empty vesicles displayed an average size of about 65 nm but did not present regular contours. On the other hand, when the vesicles were loaded with inorganic nanoparticles (i.e., QDs and MNPs), their structure became more regular with an evident turgidity and three-dimensionality. This effect could be caused by the evaporation of the solvent during TEM sample preparation, with a different outcome depending on the inner lipid support structure. In fact, while empty vesicles are free to shrink while drying, the presence of inorganic nanoparticles inside the core provides a physical resistance to the contraction. It is likely that both QDs and MNPs accommodate inside the lipid bilayer because of their hydrophobic nature (thanks to the alkyl chains of the surfactants covering the inorganic core). However, the thickness of such layer should be smaller than 4 nm considering that the phospholipids have an alkyl chain of 12 carbon atoms, while the average core diameter of the QDs and of the MNPs is around 4 and 6 nm, respectively, as determined by TEM analysis (Figure S1). Moreover, both inorganic NPs are coated by surfactant molecules that would further increase their size of about 1 nm. As a result, the dimension of the inorganic particles should exceed significantly that calculated for the double layer. Despite this, both QDs and MNPs were successfully incorporated in the NVs, probably because of the flexibility of the lipid bilayer. Indeed, other studies already reported the encapsulation of inorganic nanoparticles with size comparable or even larger than that of the bilayer thickness. QDs, for instance, were incorporated into the lipid shell because of such flexibility, leading in some cases to the formation of small protrusions (Al-Jamal et al., 2008; Bothun et al., 2009; Kethineedi et al., 2013).

TEM images of the vesicles encapsulating the nanocrystals showed regular and uniform morphology with an average diameter of about 104 nm in the case of QD-loaded NVs, and of about 125 nm for the magnetic ones (Table 2; see also Figure S4 for the histograms of the size distribution). Figure 3D shows the morphology of the lipid vesicles loaded with the organic DOP-F-DS fluorophore, whose average size is about 67 nm, comparable to that of empty liposomes: it is highly plausible that its alkyl chain is situated into the lipid bilayer (Cardone et al., 2012). The image of panel E refers to the vesicles loaded with transferrin-TRICT (a protein with 80 kDa molecular weight): the hydrophilic nature of the protein suggests an accommodation into the aqueous core, leading to an enlargement of the vesicle diameter up to 78 nm. The same sample was imaged after 2 weeks of storage in PBS at 4°C (Figure S2): size and the of the vesicles were retained but it can be denoted the presence of debris, material depots on the grid bed that may be due to an initial degradation of the lipid texture. Panel F refers to the vesicles loaded with both molecules yielding slightly enlarged NVs compared to those encapsulating either DOP-F-DS or transferrin.

**Table 2:** Average size of the lipid vesicles, either empty or loaded with different molecules, estimated through Image J Software on TEM images of the particles (second column), and by DLS analysis (third column).

**Lipid vesicles**	**Average size from TEM images (nm)**	**Average size from DLS (nm)**	**PDI**	**Surface charge (mV)**
Empty	65 ± 8	137 ± 11	0.27	−4.9 ± 0.3
QDs (4 nm size)	104 ± 13	278 ± 21	0.29	−5.7 ± 0.6
MNPs (6 nm size)	125 ± 12	319 ± 24	0.34	−4.8 ± 0.8
DOP-F-DS	67 ± 10	148 ± 8	0.22	−5.2 ± 1.1
Transferrin-TRITC	78 ± 7	207 ± 13	0.28	−8.9 ± 0.7
DOP-F-DS + Transferrin-TRITC	88 ± 9	298 ± 19	0.22	−6.0 ± 1.1
Doxorubicin	71 ± 7	187 ± 21	0.33	−5.3 ± 0.3
SN-38	66 ± 7	163 ± 14	0.20	−5.4 ± 0.1
Doxorubicin and SN-38	103 ± 8	245 ± 9	0.21	−5.8 ± 0.2

*The fourth column reports the polydispersity index (PDI), while the last ones shows the values of surface charge for each type of vesicles*.

Next, two chemotherapeutic drugs, namely doxorubicin and SN-38, being the former dispersible in water while the latter poorly soluble and instable at physiological pH, were encapsulated into the vesicles. Figures 3G–I report the TEM images of the liposomes loaded with doxorubicin, SN-38, and both drugs, respectively. The size of the vesicles loaded with the individual drugs was around 70 nm in both cases, while the particles simultaneously encapsulating the two molecules displayed an average size of around 100 nm (see Table 2). As already mentioned, it was necessary to prolong the sonication time to 6 min when preparing vesicles simultaneously loaded with doxorubicin and SN-38. Indeed, after 4 min (that is the time needed for preparing all the other formulations) the vesicles had not formed yet, while closely attached lipid droplets of various sizes could be observed by TEM (Figure S3), evidencing the incomplete formation of the spherical vesicles. The need for prolonged sonication time might be attributed to the interaction between the two drugs that might influence bending and sealing of the bilayer.

The analysis of the hydrodynamic diameter of the vesicles (third column of Table 2 and Figure S4) evidenced a broadening of the size when the particles were hydrated and dispersed in PBS. As expected, the size of the empty vesicles (137 ± 11 nm) was smaller compared to that of the loaded vesicles, as already observed by the TEM analysis. The largest particles were those loaded with both drugs (245 ± 9 nm), QDs (278 ± 21 nm), DOP-F-DS, and transferrin-TRITC (298 ± 19 nm), and the magnetic nanoparticles (319 ± 24 nm). The polydispersity index (PDI) values ranged between 0.2 and 0.3 and the suspensions resulted to be very stable over time, as confirmed by DLS measurements after 7 and 14 days (samples were stored at 4°C but they were kept for 1 h at room temperature before measurement). As shown in Table S1, the size of the empty vesicles and of those loaded either with the single drugs or with both doxorubicin and SN-38 remained constant and the suspension did not look altered even after 2 weeks.

The average surface charge of the vesicles was slightly negative (about −6 mV) for all the different formulations prepared, independently of the loading or the dimension of the NVs (Table 2).

Incorporation of the MNPs into the NVs conferred a magnetic behavior to the loaded NVs. As a proof of concept, a magnetic field (0.3 T) was applied through a magnet close to the wall of a vial containing a solution with the NVs. As expected, the nanovesicles adhered to the wall adjacent to the magnet within 2 h (Figure S5). The process was shown to be reversible and repeatable.

**Figure 4:**
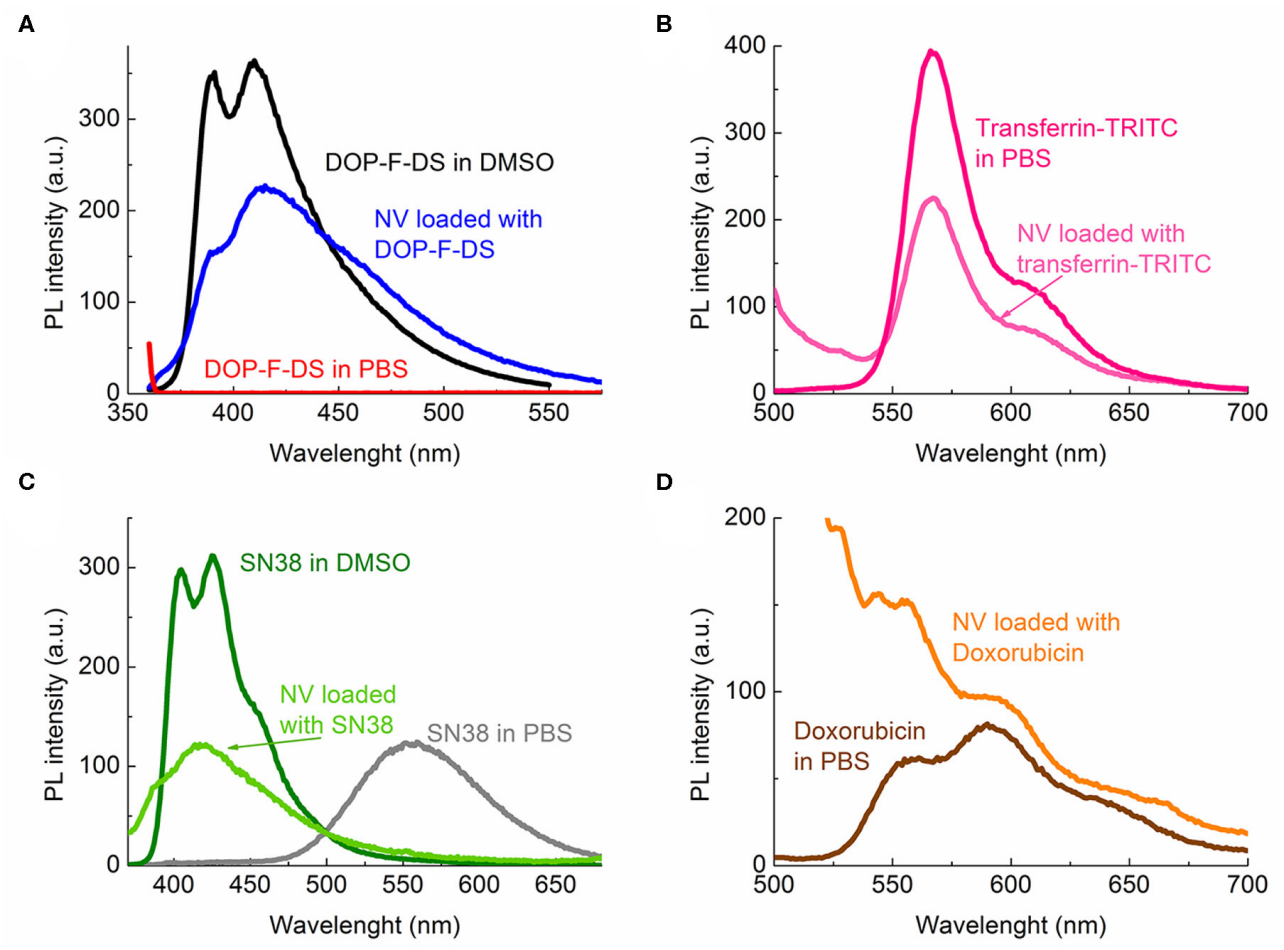
Photoluminescence spectra of the fluorescent species either free or encapsulated: **(A)** DOP-F-DS; **(B)** transferrin-TRITC; **(C)** SN-38; and **(D)** doxorubicin.

To confirm whether the different species were encapsulated in the bilayer or within the core, the fluorescence spectra of the loaded vesicles were collected. Figure 4A) shows the emission spectra of the organic DOP-F-DS fluorophore dissolved in DMSO (black curve), in PBS (red curve), and in the vesicles suspended in PBS (dark blue curve). The fluorescence curve of the encapsulated dye is quite similar to that of the free dye in DMSO, showing the same two peaks (at 390 and 410 nm) although slightly broadened when encapsulated. On the other hand, the DOP-F-DS fluorescence quenches completely in PBS, indicating that the dye most likely settles into the double layer when encapsulated. As shown in Figure 4B, the fluorescence spectrum of transferrin-TRITC loaded into the vesicles is comparable to that of the free protein dissolved in PBS.

SN-38 is poorly soluble in aqueous media; indeed, the fluorescence curve of the molecule dissolved in PBS was considerably different from that in DMSO (Figure 4C). In this solvent (dark green curve), the spectrum displayed two peaks at 405 and 425 nm, respectively; while in PBS (gray curve) the fluorescence shifted to 555 nm, likely due to spatial arrangement and π-π interactions among the molecules. On the other hand, the fluorescence curve (light green) was slightly shifted toward higher energies when it was encapsulated into the vesicle, with the two peaks detected at 387 and 417 nm, respectively. Therefore, it is reasonable to suppose that, similarly to the DOP-F-DS dye, SN-38 is also hosted into the lipid bilayer of the vesicles.

The main emission peak at 595 nm of free doxorubicin in PBS (dark red curve) was retained when encapsulated into the vesicles (orange curve), as shown in Figure 4D. The fluorescence spectrum in PBS of the nanovesicles loaded with the green-emitting QDs was also detected (Figure S6) and the obtained curve was similar to that of the free surfactant-coated nanocrystals. However, a slight broadening of the maximum peak was noted, again suggesting encapsulation of the QDs into the lipid bilayer, as also expected since they are not soluble in aqueous medium and their fluorescence would be quenched in this solvent.

### Drug Loading and Release From the Lipid Nanovesicles

The optical spectra of doxorubicin and SN-38 were used to monitor the amount of drug incorporated into the NVs and to calculate the encapsulation efficiency (*EE*). As expected, *EE* for doxorubicin was dependent on the concentration of the feeding solution, reaching a maximum of about 6% when using an 85.8 μM initial solution. *EE* dropped to 3.5 and 2.7% when using lower initial concentrations (42.9 and 21.5 μM, respectively). On the other hand, higher *EE* values were generally obtained with SN-38, although a decreased efficiency was observed (40, 30, 20, and 15%) by increasing the concentration of the feeding solution (0.5, 1, 2, and 3 μM, respectively).

Two parameters must be considered to explain the different behavior of the two drugs, i.e. the different solubility and the preparation conditions of the vesicles. SN-38 has a poor solubility in PBS and tends to intercalate spontaneously within the double layer given the greater affinity for the lipophilic environment. On the other hand, doxorubicin is perfectly soluble in PBS and the encapsulated amount mainly depends on the maximum capacity of the vesicle core.

**Figure 5:**
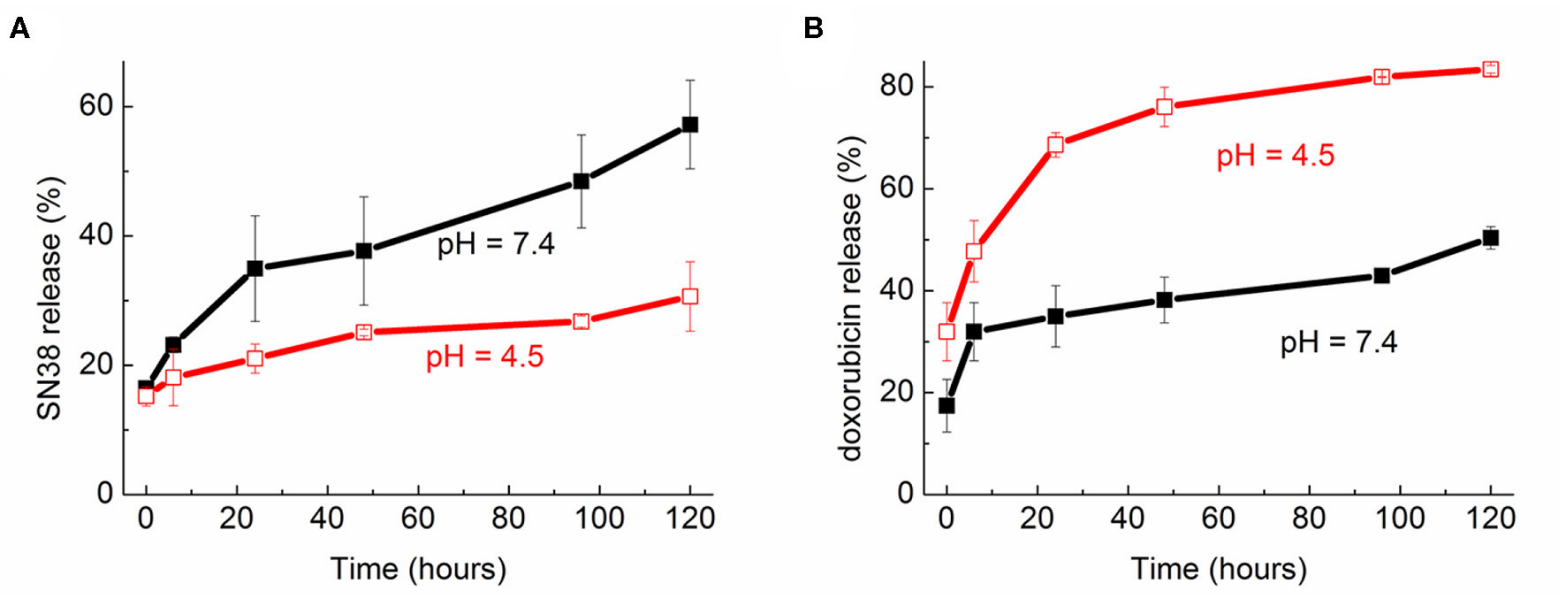
Release profile over time (up to 120 h) of **(A)** SN-38 and **(B)** doxorubicin from the nanovesicles at two pH (7.4 and 4.5).

The *in vitro* release kinetics of the two drugs based on the NVs with the highest *EE* were also studied. As reported in Figure 5, the release was monitored up to 5 days keeping the samples at 37°C and at two pH values, 7.4 and 4.5, the latter being resembling the condition in the intracellular endolysosomes. As expected, the different solubility of the two drugs also led to completely different patterns. In the case of SN-38 the highest release was observed at physiological pH, reaching 35% after 24 h and 57% after 120 h. The release resulted considerably reduced at lower pH, reaching a maximum of about 30% after 5 days.

On the other hand, there was a quicker and more significant release of doxorubicin at acidic conditions as compared to physiological ones. Indeed, after 24 h incubation at 37°C the release was around 35% at pH 7.4 and almost doubled (68%) at pH 4.5. The highest release was detected after 5 days in acidic solution, reaching 83% of the total encapsulated amount.

### Cellular Studies

#### Viability Assays

Cellular viability studies were performed to assess the effectiveness of the NVs loaded with the chemotherapeutic agents and to evaluate the effects of the empty ones. Two human cell lines derived from breast cancer, MDA-MB-231, and MCF-7, were used.

Two viability assays, namely the metabolic MTT and the Trypan blue exclusion assay, were performed administering the cells with the vesicles encapsulating the drugs, either alone or simultaneously. Additionally, the viability of the cells incubated with the free drugs was also evaluated. Based on our previous studies, the drug amount administered to the cells was fixed to 0.5 μM for doxorubicin and 0.1 μM for SN-38 (Deka et al., 2011; Elbialy and Mady, 2015; Fang et al., 2018). These values are consistent with average IC_50_ values from the literature for MDA-MB-231 and MCF-7 cells, being between 1.7 and 0.2 μM that for doxorubicin and between 0.3 and 0.02 μM that for SN-38 (https://www.cancerrxgene.org/; Jandu et al., 2016; Lin et al., 2019).

**Figure 6:**
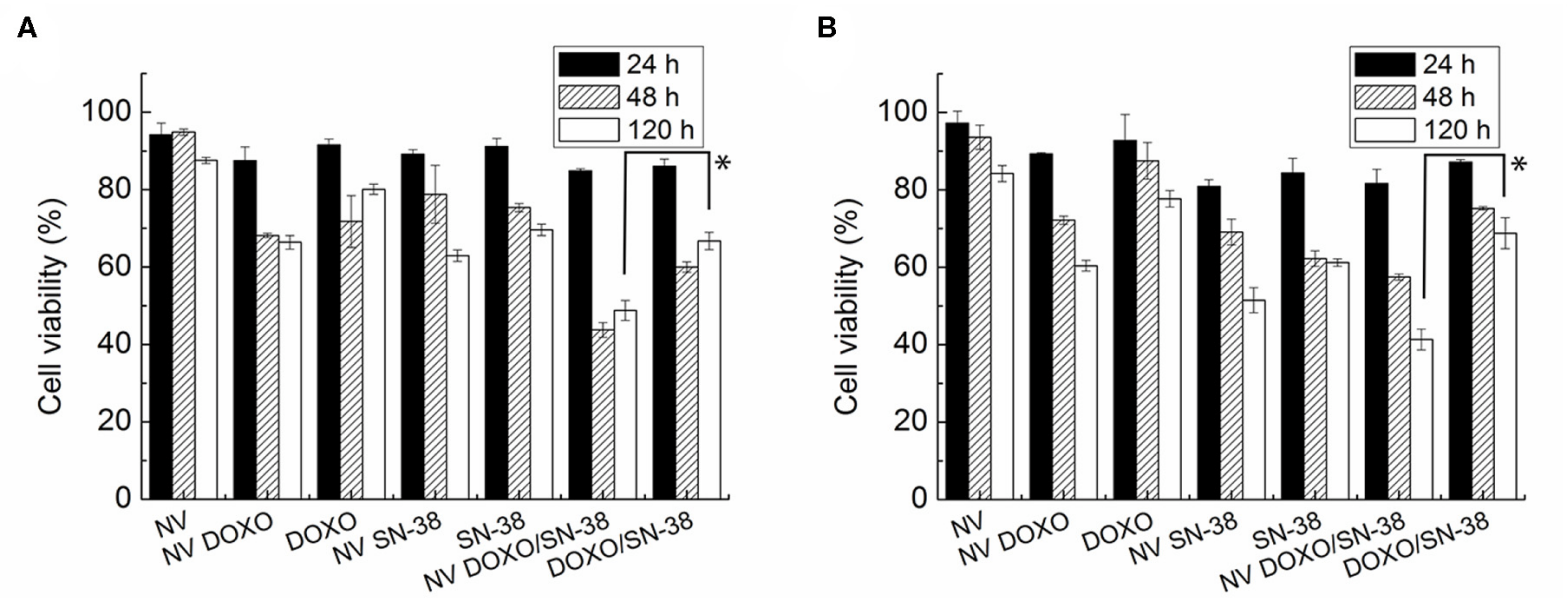
MTT viability assay of **(A)** MDA-MB-231 and **(B)** MCF-7 cells administered for 24, 48, and 120 h with empty nanovesicles, nanovesicles loaded with DOXO, free DOXO, nanovesicles loaded with SN-38, free SN-38, nanovesicles loaded with both drugs, and both free drugs, respectively. The viability of the cells incubated with the loaded nanovesicles was compared with that of the cells incubated with free drugs, at 120 h in both cell lines. Statistical analysis was performed via *t*-test considering it significant for *p < 0.05.

As evidenced in Figure 6, the empty NVs displayed negligible toxicity according to the MTT assay, reaching a viability close to 80% after 5 days. At the administered drug concentration, the nanovesicles loaded with doxorubicin led to a reduction of viability up to 60% in both cell lines after 120 h, while the toxic activity of the free drug resulted to be very limited even after 5 days, with viability values close to 70%. In the case of SN-38, after 24 h the viability of both cell lines dropped to 70% when encapsulated, while it settled at 80% when administered as free molecule.

Encapsulation of the drugs into the vesicles increased significantly their effectiveness, likely thanks to a facilitated uptake from the cells. This effect was enhanced when both drugs were encapsulated within the vesicles, and it became more pronounced with increasing incubation times. Indeed, the combined toxic effect led to a reduction in cell viability to values below 50% after 48 h in both cell lines, and down to 26 and 22% after 5 days in MDA-MB-231 and MCF-7, respectively. On the other hand, when doxorubicin and SN-38 were administered simultaneously to the cells as free formulation (at the same concentrations loaded into the NVs), viability reached values close to 75 and 45% after 24 and 120 h, respectively. The increased toxicity of the vesicles loaded with both drugs became statistically significant (*p* < 0.005) in both cell lines only after long incubation time (5 days). These findings suggest that when the encapsulated drugs are internalized by the cells, they are not immediately available to exert their activity but they are released gradually, as confirmed by the drug release kinetic showed in Figure 5. The limited activity shown by the combination of the free drugs probably depend on the partial degradation of the molecules, that is prevented when they are encapsulated, as well as on the existence of several drug resistance mechanisms in breast cancer cells, as already reported (Jandu et al., 2016). These effects, (i.e., the protective role of the NVs, the facilitated internalization, and the sustained release, show the advantage of using the vesicles for delivering the drugs). However, from Figure 6 it is evident that the pharmacological activity of the drug loaded NVs is poor in the first 48 h post administration. Several factors, such as surface chemistry of the NVs, physico-chemical properties of encapsulated drugs, and cells used, can contribute to this effect thus affecting internalization of the carrier, drug release kinetic, and activation of drug efflux pumps. To determine whether the NVs loaded with both drugs induced a synergic cytotoxic effect, the CI was calculated according to the Chou and Talalay's formula (Chou and Talalay, 1984). A value of 0.97 was obtained, suggesting a slight synergic effect.

The data obtained by the vital Trypan blue assay (Figure S7) are in agreement with those obtained by the MTT assay, confirming a progressive reduction of the number of viable cells upon incubation with the nanovesicles loaded with the drugs, reaching the highest mortality when both drugs were hosted into the lipid vesicles. Indeed, the toxicity of the NVs loaded with both drugs is statistically significant (*p* < 0.005) in both cell lines after 120 h. This assay also confirmed the biocompatibility of the empty vesicles, that did not impact the number of vital cells; their number, in fact, remained close to that of the control samples after 24 and 48 h, and it still maintained values above 80% after 5 days in both cell lines.

It is worth reminding that the cellular target of doxorubicin and SN-38 is the nucleus. Although their mechanism of action is not fully understood yet, they both inhibit DNA replication and translation, doxorubicin by intercalating into DNA, and inhibiting topoisomerase II, while SN-38, the active form of irinotecan, binds to the topoisomerase I-DNA complex (Kawato et al., 1991; Yang et al., 2014). The results obtained by the cellular assays presented in this section show that the drug-loaded NVs affect cell viability of the two cell lines, but they do not shed light on the cellular pathways and the molecular mechanisms involved in this process. Deeper investigation of these effects deserves further studies that, however, are beyond the aims of this work.

#### Cellular Internalization

To elucidate the internalization process of the nanovesicles and their intracellular localization, cells doped with NVs loaded with fluorescent molecules were analyzed by optical imaging.

**Figure 7:**
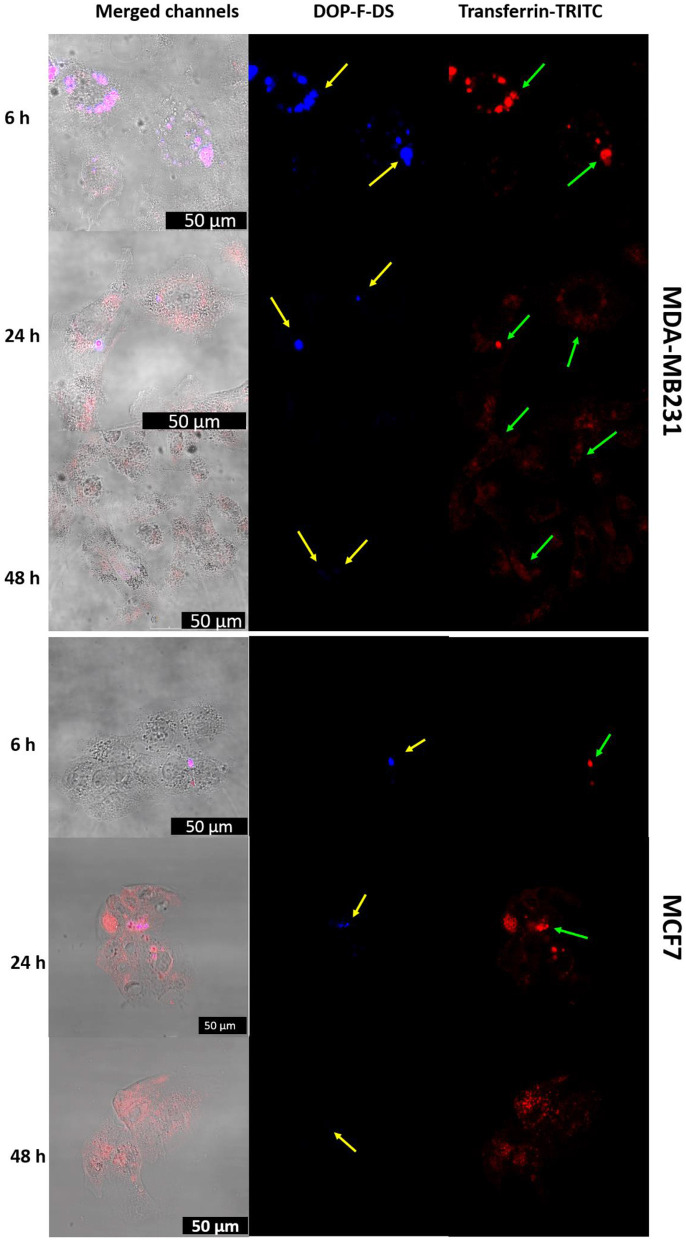
CLSM images of MDA-MB-231 and MCF-7 cells incubated with liposomes loaded with transferrin-TRITC and DOP-F-DS for 6, 24, and 48 h. The left column refers to the fluorescence channels merged with the bright field, while the central and right columns refer to the blue and red fluorescence channels, respectively. The scale bars correspond to 50 μm.

In detail, MDA-MB-231 cells were incubated with NVs hosting the DOP-F-DS fluorophore, contained in the lipid bilayer, and the transferrin-TRITC protein, hosted into the vesicle core. After 6, 24, and 48 h the cells were fixed and imaged by CLSM (Figure 7, upper panels). The vesicles were already internalized and located into the perinuclear endosomes after 6 h. Complete overlap between the DOP-F-DS signal (blue channel) and the transferrin-TRITC signal (red channel) could be observed, indicating that the vesicles did not yet undergo enzymatic degradation. However, a significant weakening of the fluorescence in the blue channel was observed after 24 h, likely due to initial partial degradation of the bilayer structure, with consequent release of DOP-F-DS and quenching of its fluorescence in aqueous medium. This effect was enhanced after 48 h, when the blue fluorescence almost disappeared. On the other hand, the TRITC-derived fluorescence, which was initially confined into the endosomal vesicles with the typical point-like fluorescence (after 6 and 24 h), seemed to diffuse into the cell cytoplasm after 48 h.

The release of molecules and drugs from the endosomes because of a temporary permeability of the organelle membrane is an already described phenomenon (Singh et al., 2004; Muripiti et al., 2018; Quarta et al., 2019). It can depend on many factors, such as size, molecular weight, charge, and solubility of the molecules that diffuse out and it is triggered by environmental parameters, such as pH and the presence of charged species.

The same CLSM experiment was also performed on MCF-7 cells, confirming the same internalization pathway (Figure 7, lower panels). However, despite the similar behavior, internalization seemed to be improved with the MBA-MB-231 cells. After few hours, the two fluorescence signals colocalized into vesicular structures, while the red signal of transferrin started to spread into the cytosol and the blue fluorescence tended to disappear after 24 h.

To confirm the intracellular compartmentalization of the NVs, a colocalization study of the NVs loaded with Transferrin-TRITC was performed with LysoTracker Green in MDA-MB-231 cells (Figure S8). Noteworthy, the distribution of the red fluorescence signal of transferrin changed over time: after 6 h some orange spots (due to the overlap of the two fluorescent signals) were visible in the perinuclear region of neighboring cells. After 24 h incubation, the endolysosomes that entrapped the NVs seemed enlarged. Then, after 48 h, the red fluorescence of transferrin started to spread into the cytoplasm (a red background was visible inside some cells) due to its release from the endolysosomal compartment.

On the other hand, when the cells were incubated with the QD-loaded nanovesicles, the fluorescence spot was localized into the endosomes at all the analyzed time points. As shown in Figure S9, the nucleus of the cells was labeled with DAPI while the cytoskeleton with phalloidin-TRITC, and the fluorescence signal of the QDs could be detected into perinuclear endosomes even after 48 h. The detection of fluorescence spots after 2 days might be due to continuous uptake of new nanovesicles inside the cells. In fact, once internalized into the endolysosome compartments, the NVs are probably slowly digested leading to fluorescence quenching. Indeed, the QDs, coated only by the lipophilic surfactant layer, are neither soluble nor stable in aqueous environment and their fluorescence is detectable until the vesicles remain intact, while it is quenched when the lipid bilayer is degraded. Moreover, it should be observed that the fluorescence intensity of the QDs signal did not increase over time inside the cells, as instead observed in the case of transferrin-TRITC (due to the progressive internalization of the NVs).

#### Lipidomic Analysis

To better understand the behavior of the drugs and the influence of the vesicular formulation, a metabolomic study was performed through ^1^H NMR on the lipid extracts obtained from the MDA-MB-231 cells. Analysis were performed on the cells treated for 24 h with doxorubicin and SN-38 either free or administered through the nanovesicles. MDA-MB-231 extracts of the untreated cells were taken as control reference. The study was performed after 24 h of incubation, as confocal microscopy imaging and MTT assay already showed noteworthy internalization and physiological effect of the drugs after this time; while MDA-MB-231 cells were considered, as NVs internalization was shown to be greater with this cell line compared to the MCF-7 (see Figure 7). Multivariate statistical analysis (MVA) was then performed on the registered NMR spectra to check if any differences could be observed between the two types of drug administration. This approach has already proved to be a valuable tool for understanding the mechanism of action of drugs and how they influence normal physiological patterns, as well as for discriminating healthy cells from cancerous ones, both *in vitro* and *ex vivo/in vivo*, thus paving the way toward personalized medicine (Armitage and Southam, 2016; Del Coco et al., 2019; Giudetti et al., 2019).

**Figure 8:**
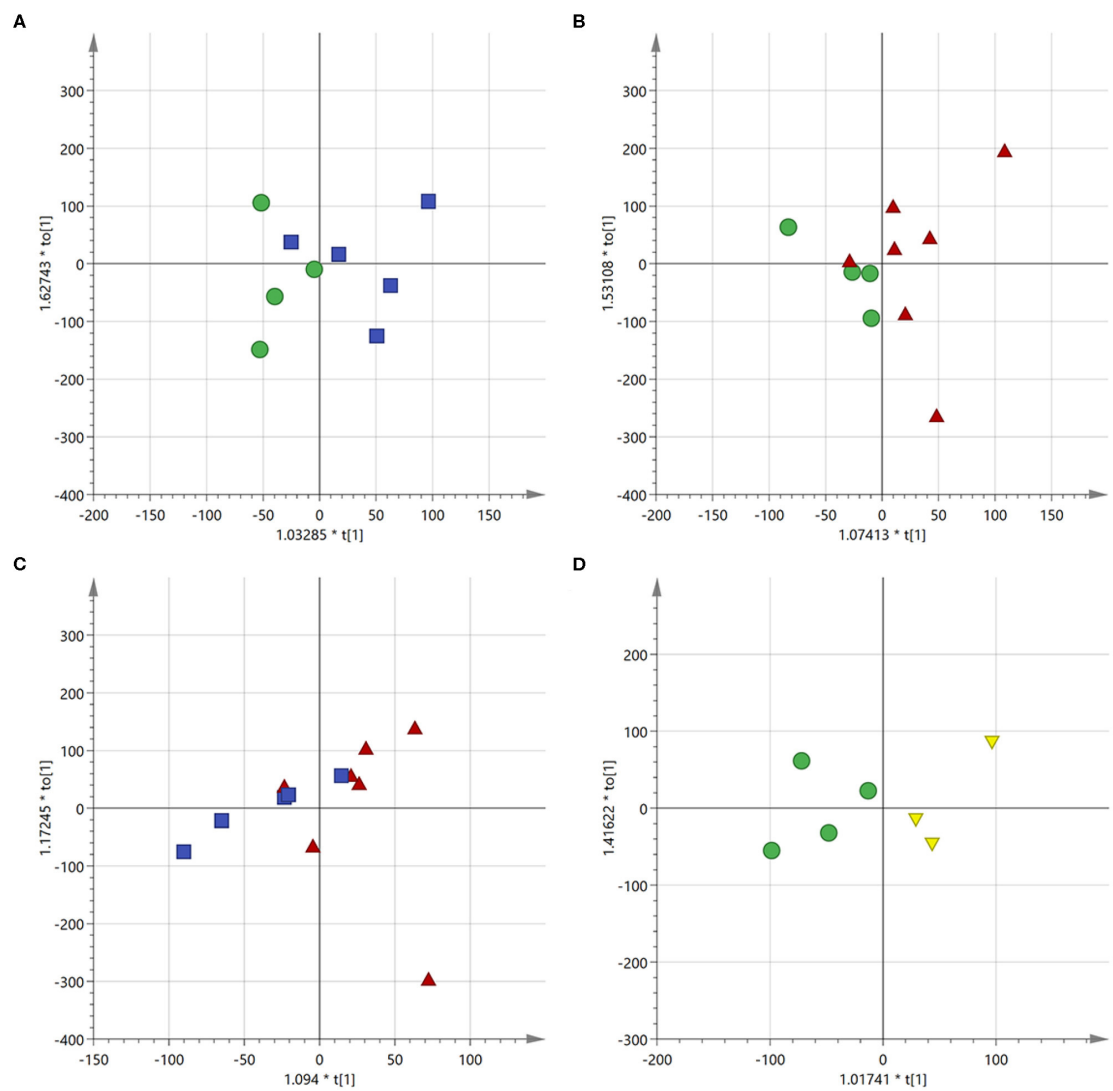
Score scatter plot for the first component of the OPLS-DA models. **(A)** Control cells (green circles) vs. free drugs (blue squares); **(B)** control cells (green circles) vs. drugs encapsulated into the NVs (red triangles); **(C)** cells treated with free drugs (blue squares) vs. those treated with the encapsulated drugs (red triangles); **(D)** control cells (green circles) vs. empty NVs (yellow triangles).

**Figure 9:**
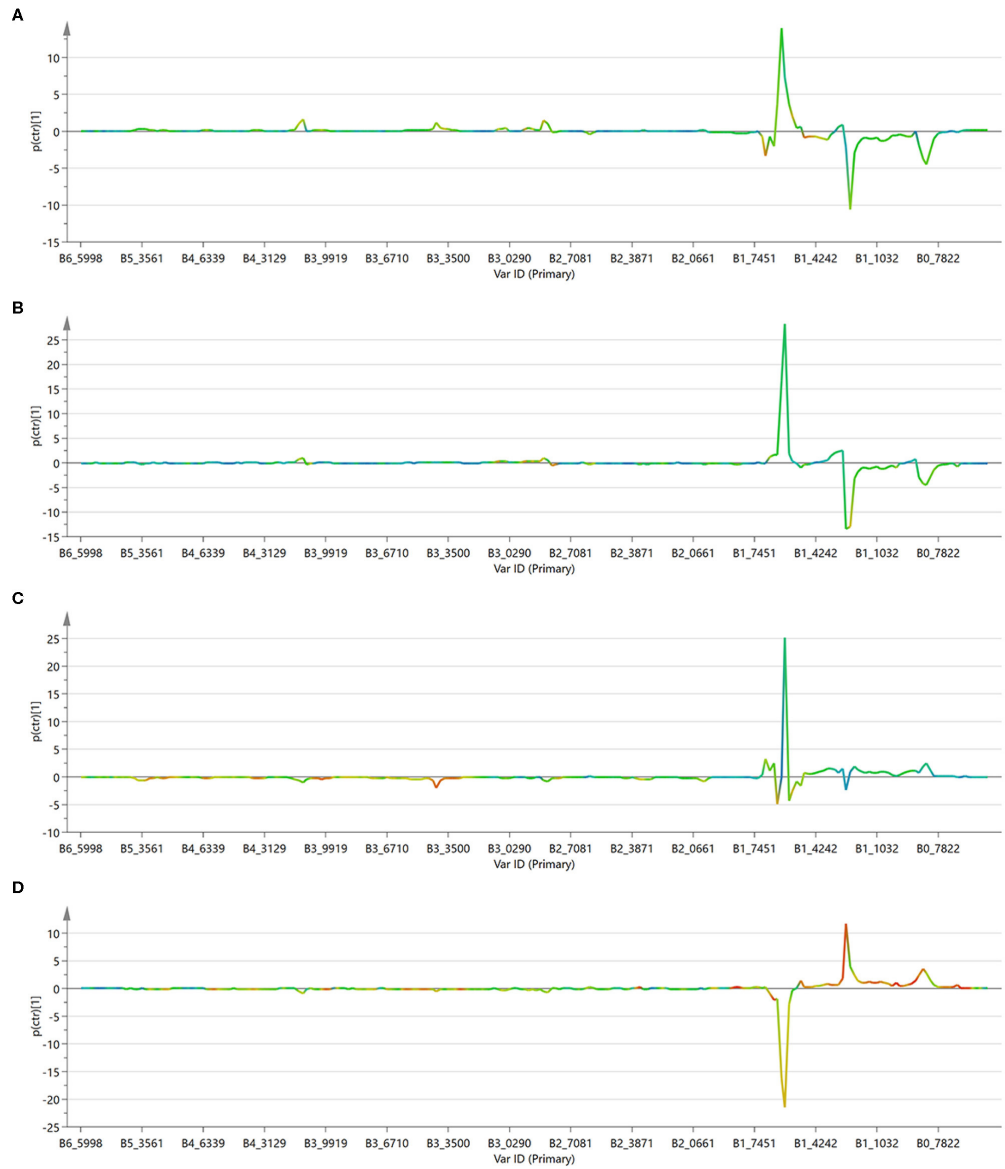
S-line plot for the first component of the OPLS-DA models. **(A)** Control cells vs. free drugs; **(B)** control cells vs. drugs encapsulated into the NVs; **(C)** cells treated with free drugs vs. those treated with the encapsulated drugs; **(D)** control cells vs. empty NVs.

The OPLS-DA model was able to discriminate between control and cells treated with either free or encapsulated drugs with a $R_{\text{X}}^{2}$ of 0.766 and 0.813, respectively (Figures 8A,B). Moreover, some differences were also observed between the two types of administration (Figure 8C). Treatment of the MDA-MB-231 cells with the two types of drug administration induced similar metabolic changes in the control cells, as observed in the profiles of the corresponding *S*-line plots (Figures 9A,B). The drugs caused a significant reduction of the total fatty acids (peaks at about 0.8 and 1.2 ppm) and a concomitant increase of the unsaturated fatty acids (peak at about 1.6 ppm). The peak at about 2.8 ppm, corresponding to the *bis*-allylic protons of polyunsaturated fatty acyl chains, and that one at 4.1 ppm, relative to vinylic protons in β to the carboxylic acid of fatty acids, also showed increased intensity in treated cells, although to a minor extent compared to the previous signals. A similar behavior was already observed in cells treated with antitumoral drugs and it has been related to an increased mobility of the cellular membrane, as a consequence of the reduced tight packing of the fatty acids. Eventually, this destabilization increases with time and finally ends up with the membrane disruption, resulting in cellular death and formation of lipid droplets (Hakumäki et al., 1999; Griffin et al., 2003).

The profile of the two curves (Figures 9A,B) looks very similar, indicating that the mechanism of action of the drugs does not change significantly depending on the mode of administration. It has to be observed, however, that phosphatidylcholine derivatives (peak at about 3.4 ppm) increased more significantly in cells treated with the free drug, but not in those treated with the loaded NVs. This effect might be due to a selective release operated by the nanocarrier in particular regions of the cells and it deserves further investigation.

To better appreciate any differences between the two administration methods, the MVA was also performed directly on these two classes (i.e., free drugs and NVs, Figure 9C). This analysis confirmed what already observed by the independent comparison vs. the untreated cells, (i.e., that they induce similar effects, but it also evidenced how the amount of unsaturated fatty acids is much more abundant in the cells treated with the drug-loaded nanovesicles). This is probably due to the higher concentration of drugs able to enter the cells when administered through the nanovesicles (as already confirmed by the confocal microscopy study showing that liposomes were already internalized into vesicles after just 6 h), thus reaching more efficiently the site of action.

As a further control, untreated cells were compared to cells administered with empty NVs, thus allowing to confirm the observed cytotoxic effect is due exclusively to the drugs (Figures 8D, 9D). Being just composed of cholesterol, ceramide, and phospholipids it is not expected that this type of vectors would induce any cytotoxicity, at least at the administered concentration. In fact, cells doped with the empty NVs showed to have higher amounts of cholesterol and total fatty acids (peaks at 0.8 and 1.2 ppm), but lower quantities of unsaturated ones (peak at 1.6 ppm), as observed in the corresponding *S*-line plot (Figure 9D) which is quite specular to those obtained in cells after drug administration (Figures 9B,C).

The use of NVs thus leads to a higher efficacy of the treatment, and the efficiency of the system is even more remarkable if we consider the protection of the drugs against degradation and the cumulative therapeutic effect of the sustained release over 5 days.

## Conclusions

As witnessed by the number and relevance of publications in the field, lipid-based nanostructures still gain great attention thanks to their biocompatible and degradable nature, and to their similarity with the vesicles released by the cells in physiological and pathological conditions. Despite the high production cost and the evidence of interactions with the immune system upon *in vivo* injection, several liposomal formulations, such as Onyvide and Doxil, were approved for therapeutic use in cancer.

In this study, lipid nanovesicles composed of phospholipids, cholesterol, and ceramide with an average size of 100 nm were prepared via ultrasonication method. Several species with completely different characteristics were encapsulated into the vesicles, including a small amphiphilic organic fluorophore (DOP-F-DS), a 60 kDa protein, lipophilic (SN-38), and hydrophilic (DOXO) drugs, and also fluorescent, and magnetic inorganic nanoparticles. The morpho-structural features and the thermal properties were studied, showing that this system presents high flexibility and good stability. Viability studies and lipidomic analysis demonstrated that the two drugs induced similar metabolic changes independently of the administration method (either free or encapsulated) but they were significantly more cytotoxic into the nanoformulation, probably because of facilitated entrance, protection against degradation, and sustained release. Imaging of the nanovesicles loaded with two fluorescent organic probes showed that, once engulfed by the cells and internalized into endosomes, the vesicles undergo gradual degradation over time with consequent release of the cargo in the cytosol, first of the lipophilic compounds (loaded into the bilayer) and then of the hydrophilic ones (loaded into the core). The data collected so far proved the excellent versatility of this system as imaging and delivery nanoformulation with potential theranostic applications. Nevertheless, further investigation is needed to optimize drug encapsulation capability (i.e., by using longer fatty acid chains as well as different lipid ratios, and to study their efficacy *in vivo*).

## Data Availability Statement

The raw data supporting the conclusions of this article will be made available by the authors, without undue reservation.

## Author Contributions

AZ and AQ designed and carried out the experiments. LBi, AC, and LBl methodology and investigation. CP and AR: data analysis, writing, and editing. All authors contributed to discussion of the results.

## Conflict of Interest

The authors declare that the research was conducted in the absence of any commercial or financial relationships that could be construed as a potential conflict of interest.
