# Supplementary material for: Lipid-Based Nanovesicles for Simultaneous Intracellular Delivery of Hydrophobic, Hydrophilic, and Amphiphilic Species
Source: Front Bioeng Biotechnol. 2020 Jul 3;8:690. doi: 10.3389/fbioe.2020.00690 (PMC7350901; doi:10.3389/fbioe.2020.00690)
Supplement: Supplementary file 1 [file Data_Sheet_1.pdf]

Supplementary Material

1 Supplementary Tables

Supplementary Table 1. Average size of the vesicles (nm) after 2, 7, and 14 days, as obtained by DLS analysis.

Lipid Vesicles	2 days	7 days	14 days
**Empty**	137 ± 11	143 ± 15	146 ± 20
**Doxorubicin**	187 ± 21	194 ± 19	189 ± 26
**SN-38**	163 ± 14	169 ± 18	172 ± 21
**Doxorubicin + SN-38**	245 ± 18	237 ± 11	241 ± 18

2 Supplementary Figures

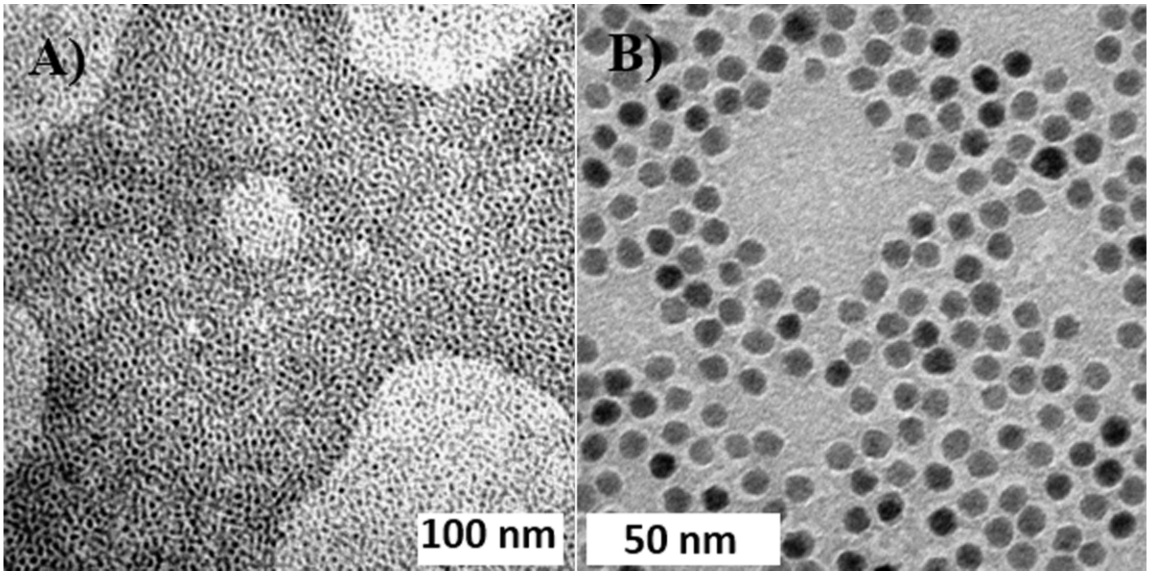

Supplementary Figure 1. TEM images of the A) QDs and B) MNPs inorganic nanocrystals encapsulated within the vesicles.

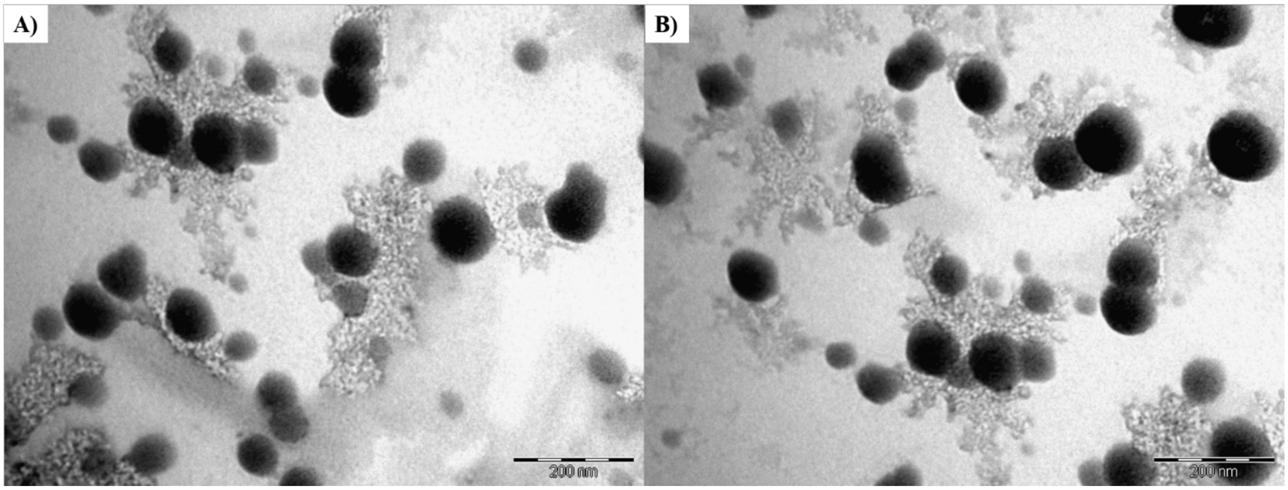

Supplementary Figure 2. TEM images of lipid vesicles loaded with transferrin-TRITC imaged after 2 weeks from the preparation.

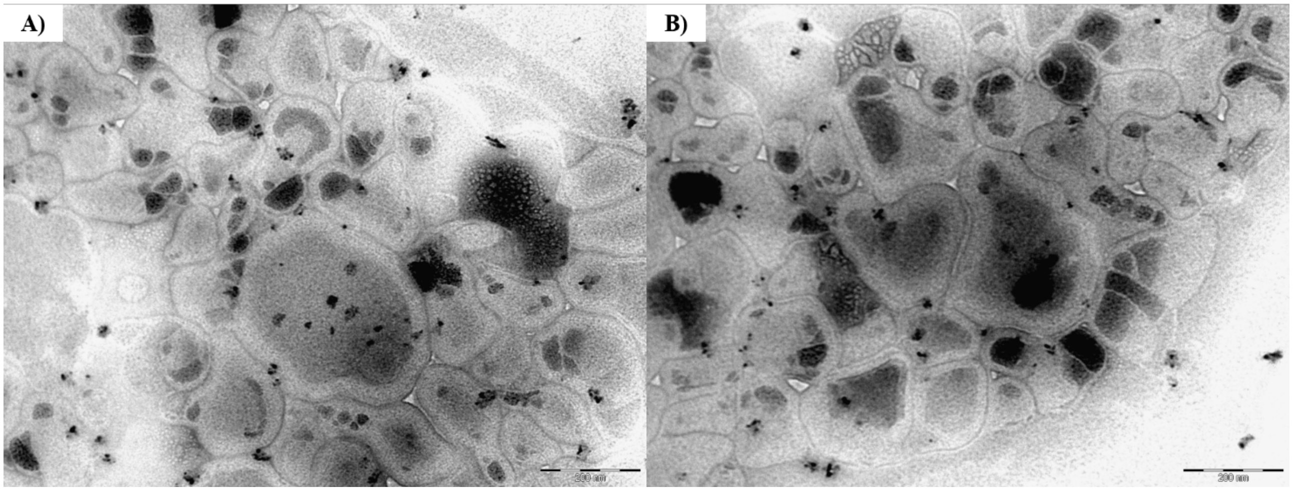

Supplementary Figure 3. TEM images of lipid vesicles loaded with SN-38 and doxorubicin, prepared with 4 minutes sonication time.

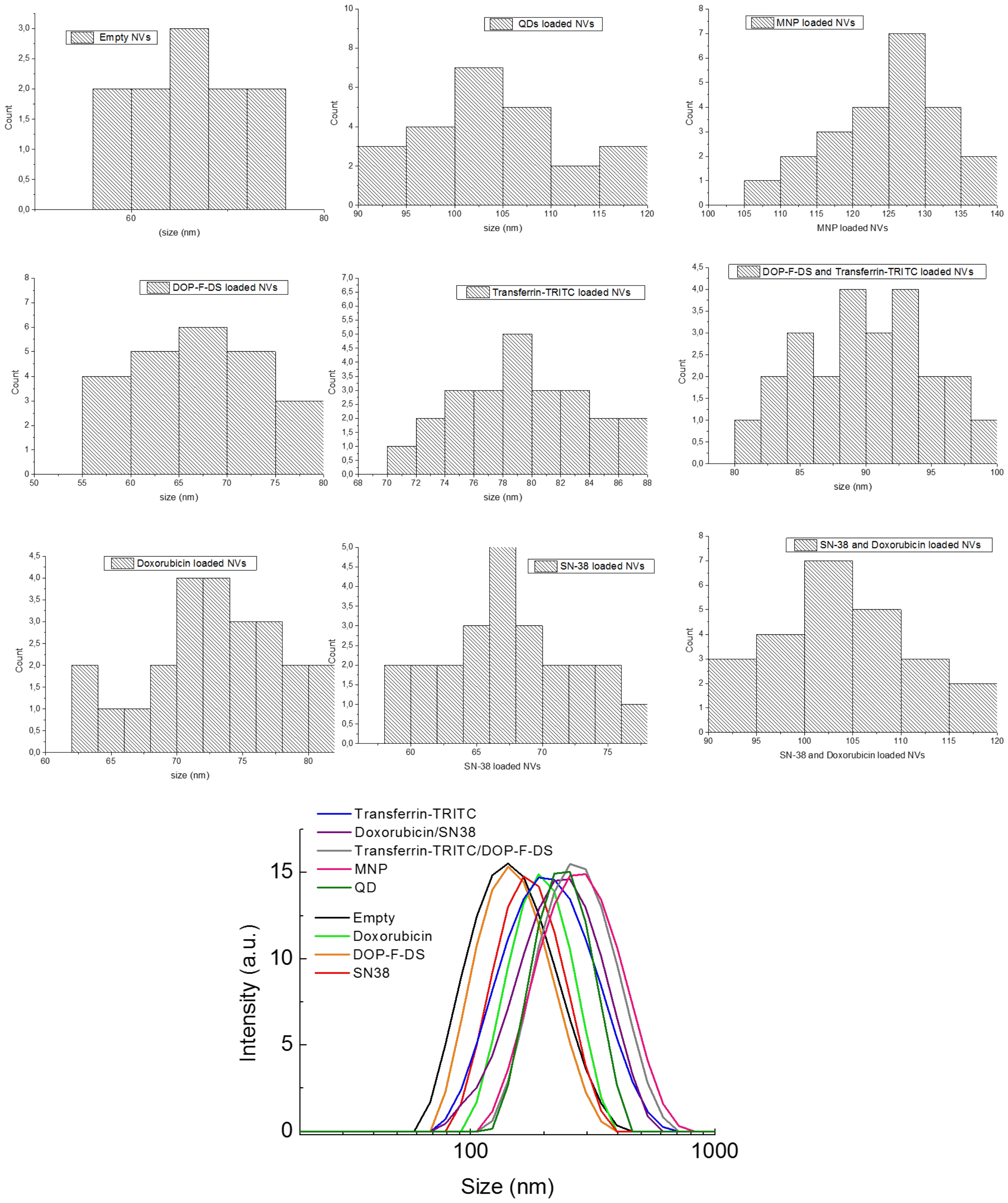

Supplementary Figure 4. Top: histograms reporting the size distribution of the nanovesicles as determined analysing the TEM images by the ImageJ software. Bottom: DLS curves reporting the hydrodynamic diameter of the nanovesicles, both empty and loaded with the different species.

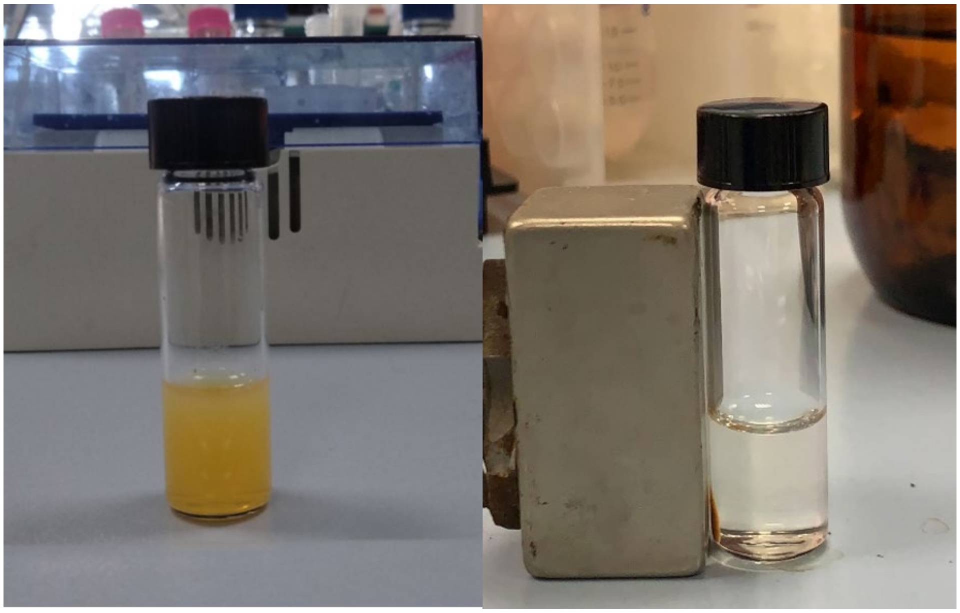

Supplementary Figure 5. Pictures of the MNP-loaded NVs solution before (left) and after (right) the application of a magnet close to the wall of the vial. The NVs were completely attached to the wall of the vial within 2 hours.

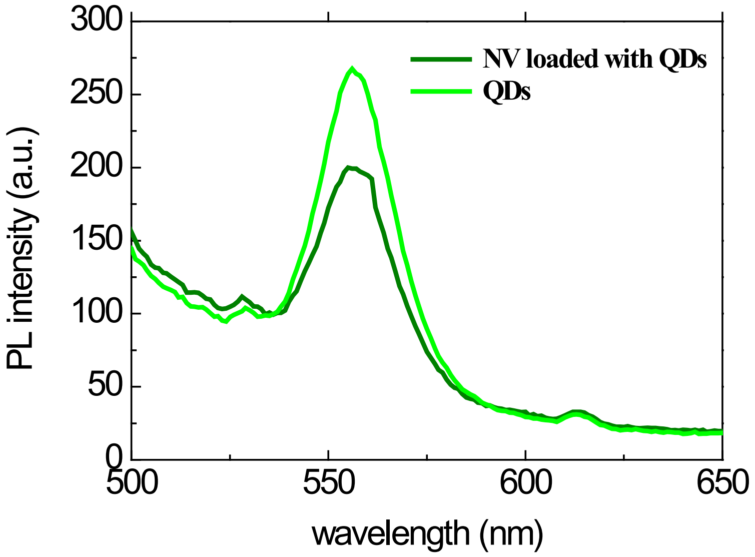

Supplementary Figure 6. Photoluminescence (PL) spectra of the green-emitting QDs either free or encapsulated into the nanovesicles.

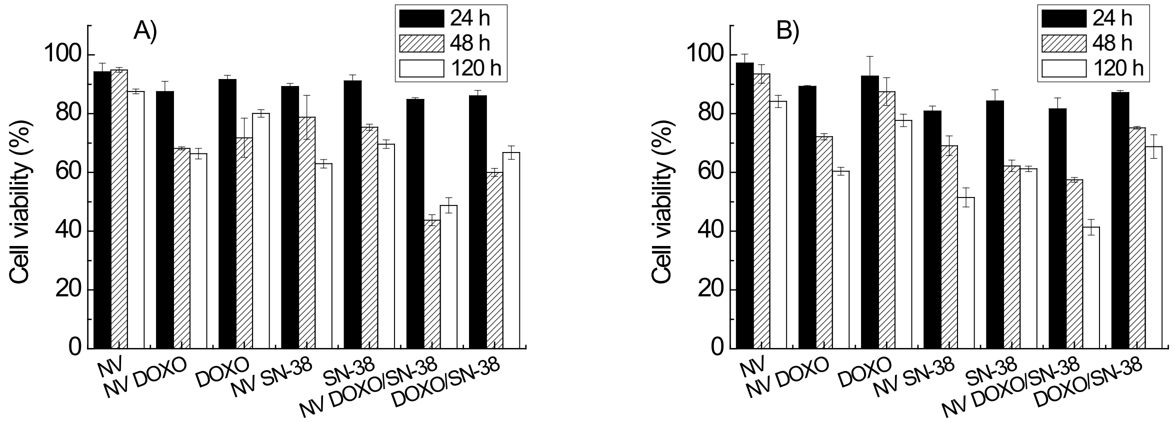

Supplementary Figure 7. Trypan blue viability assay of A) MDA-MB-231 and B) MCF-7 cells administered for 24, 48, and 120 h with empty nanovesicles, nanovesicles loaded with DOXO, free DOXO, nanovesicles loaded with SN-38, free SN-38, nanovesicles loaded with both drugs, and a mixture of both drugs.

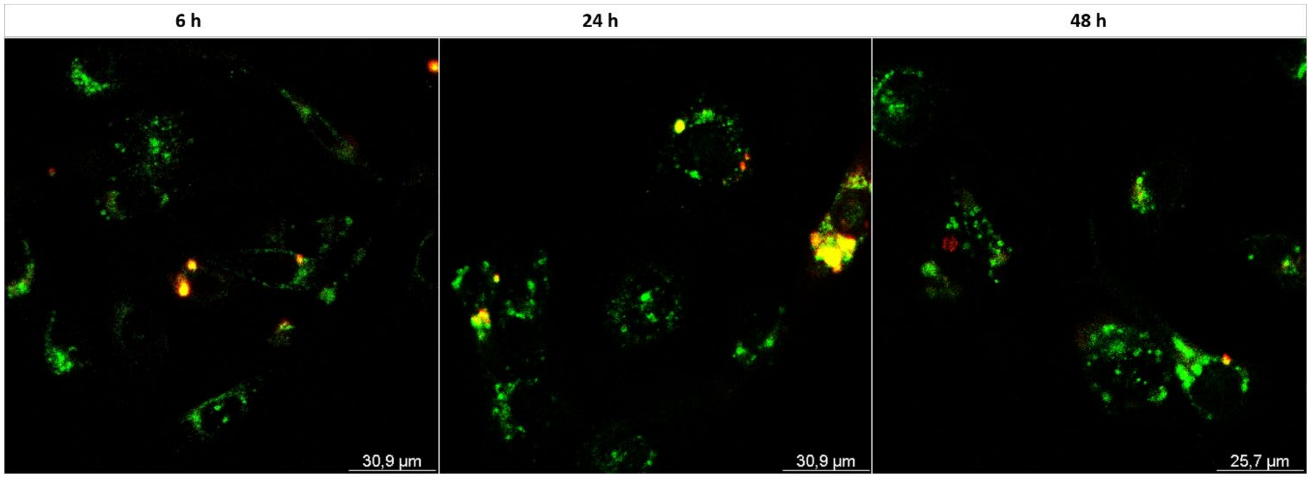

Supplementary Figure 8. Colocalization images of transferrin-TRITC loaded NVs with LysoTracker Green in MDA-MB-231 cells. The NVs were incubated for 6, 24, and 48h respectively. Images were acquired at the CLSM.

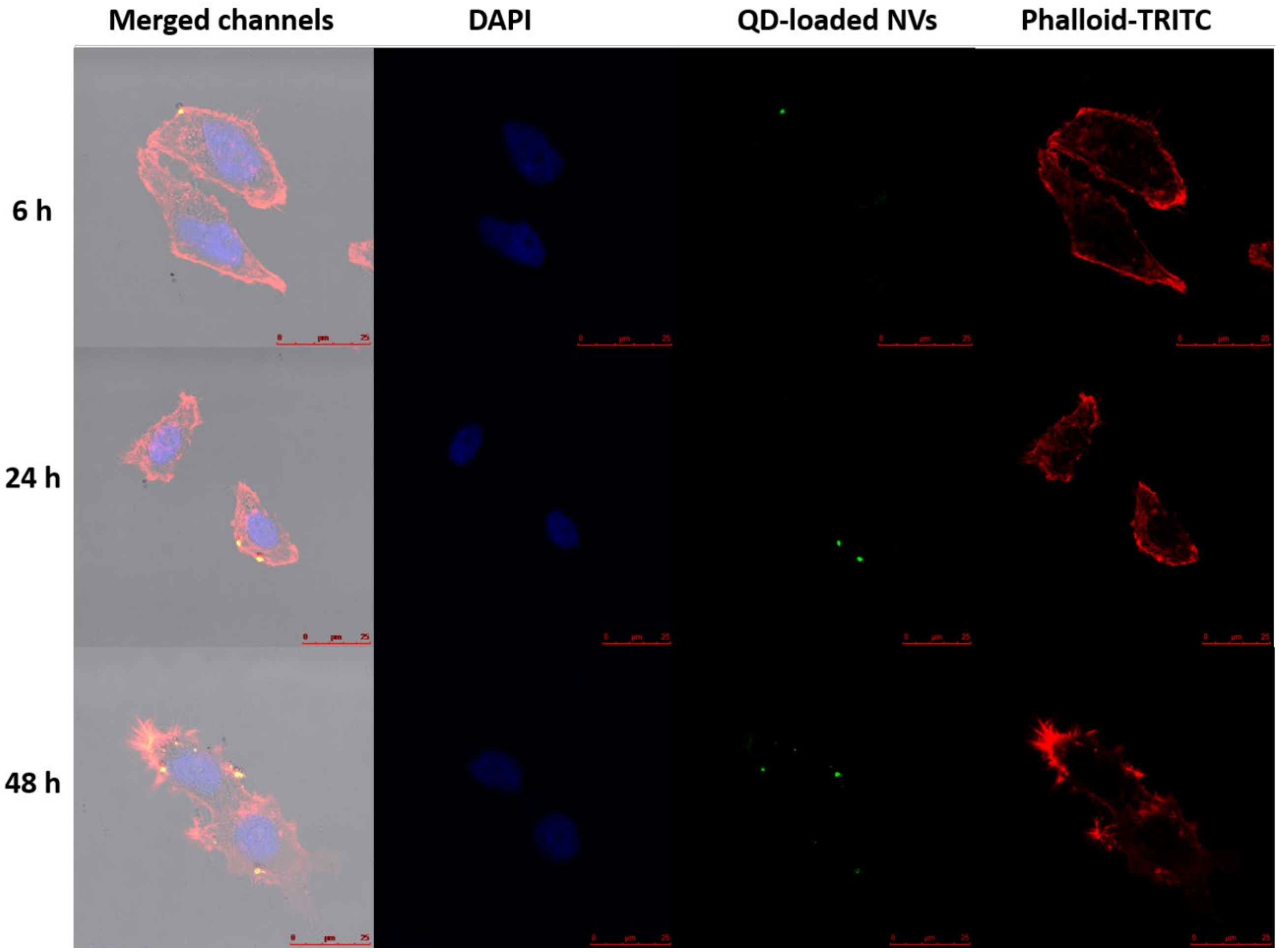

Supplementary Figure 9. CLSM images of MCF-7 cells incubated with green-emitting QDs-loaded nanovesicles for 6, 24, and 48 h. The cell nucleus was labelled with DAPI and the cytoskeleton with phalloidin-TRITC.
